# Chitosan-Coated Silver Nanoparticles Inhibit Adherence and Biofilm Formation of Uropathogenic *Escherichia coli*

**DOI:** 10.1021/acsinfecdis.3c00229

**Published:** 2024-01-29

**Authors:** Pablo Mendez-Pfeiffer, Manuel G. Ballesteros-Monrreal, Josue Juarez, Marisol Gastelum-Cabrera, Patricia Martinez-Flores, Pablo Taboada, Dora Valencia

**Affiliations:** † Departamento de Ciencias Químico-Biológicas y Agropecuarias, Universidad de Sonora, Campus Caborca, Caborca, Sonora CP 83600, Mexico; ‡ Departamento de Física, Universidad de Sonora, Campus Hermosillo, Hermosillo, Sonora CP 83000, Mexico; § Departamento de Física de la Materia Condensada, Facultad de Física, Universidad de Santiago, de Compostela CP 15782, Espana

**Keywords:** chitosan-silver nanoparticles, antibacterial activity, uropathogenic Escherichia coli, antiadherence activity

## Abstract

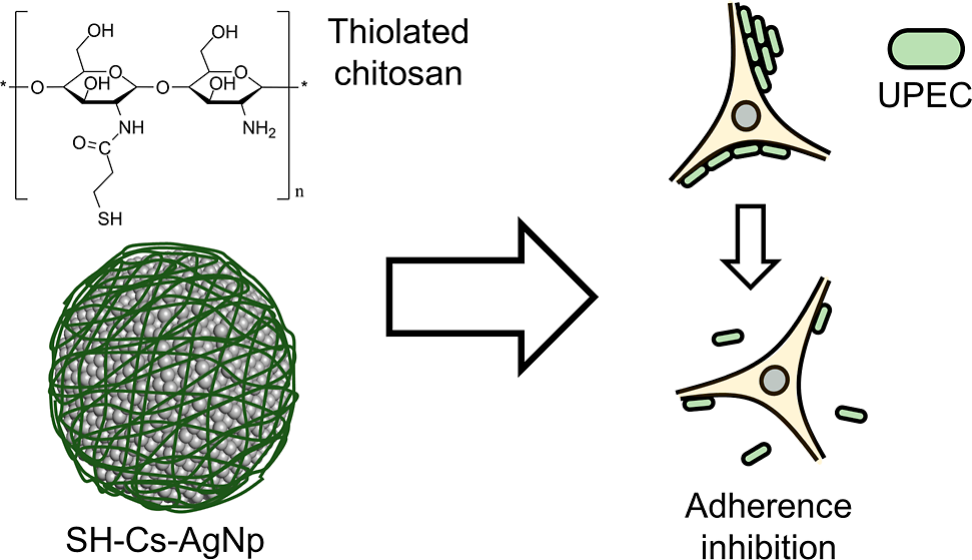

Urinary tract infections are commonly caused by uropathogenic *Escherichia coli* (UPEC), which usually presents multiple virulence and resistance mechanisms, making it difficult to treat. It has been demonstrated that silver and polymeric nanoparticles had potential against these pathogens. In this study, we synthesized thiol chitosan-coated silver nanoparticles (SH-Cs-AgNPs) and evaluated their antibacterial, antibiofilm and antiadherence activity against clinical isolates of UPEC. The SH-Cs-AgNPs showed a spherical shape with a size of 17.80 ± 2.67 nm and zeta potential of 18 ± 2 mV. We observed a potent antibacterial and antibiofilm activity as low as 12.5 μg/mL, as well as a reduction in the adherence of UPEC to mammalian cells at concentrations of 1.06 and 0.53 μg/mL. These findings demonstrate that SH-Cs-AgNPs have potential as a new therapeutic compound against infections caused by UPEC.

Bacterial infections continue to be a significant public health concern, with the emergence of antibiotic-resistant strains making treatment increasingly challenging.^1^ Uropathogenic *Escherichia coli* (UPEC) is the main causative agent of urinary tract infections (UTIs). This pathogen possesses numerous virulence factors that have enabled it to thrive in the urinary tract environment and efficiently execute its pathogenicity mechanism. Furthermore, there is a growing prevalence of multidrug-resistant (MDR) strains including carbapenemase-producing *E. coli*, which severely limit the range of available therapeutic options in the standard-of-care treatment protocol.^2,3^

For most bacterial pathogens, adherence to epithelial cells is the first step to infection and colonization.^4^ UPEC can adhere to the urinary tract epithelial cells via multiple receptors, for example, the mannosylated residues of uroplakine-1a and α3β1 integrins which are present in uroepithelial cells facing the lumen, which are recognized by UPEC’s fimbrial adhesin FimH, promoting colonization, invasion, and internalization, forming intracellular bacterial communities (IBCs), which are responsible for persistent infections, immune system evasion, and poor response to treatment.^5,6^

Therefore, the search for alternative strategies to combat bacterial infections is of the utmost importance. In recent years, nanotechnology has been explored as a potential solution, particularly the use of chitosan-coated silver nanoparticles (Cs-AgNPs) due to their unique physicochemical properties and ability to inhibit bacterial growth.^7^ Chitosan is a natural biopolymer that has been extensively investigated for its antimicrobial properties.^8^ Silver nanoparticles (AgNPs) have also been shown to possess potent antimicrobial activity due to their large surface area-to-volume ratio, which facilitates interaction with bacterial cells.^9^ Combining chitosan and AgNPs can result in synergistic effects and enhance the antimicrobial activity of both compounds.

Recent studies have evaluated the antibacterial and antibiofilm activity of Cs-AgNPs in a variety of bacterial models such as *Staphylococcus aureus*, *E. coli* and *Pseudomonas aeruginosa*; nevertheless, studies that evaluate the antivirulence activity of Cs-AgNPs are scarce.^10^ Inhibition of bacterial adherence could be an important strategy to fight bacterial infections as it prevents the development of bacterial colonization and subsequent disease progression. Nevertheless, most studies evaluate the antibacterial effect of antimicrobial agents against model or American Type Culture Collection (ATCC) bacterial strains, not considering the genetic variability of the strains that could be causing infections. Therefore, it is necessary to evaluate those antibacterial agents against clinical isolates with variable virulence and resistance characteristics.

The aim of this work was to design and synthesize a nanosystem based on chitosan-covered AgNPs and to evaluate their antibacterial, antiadherence, and antibiofilm activity against clinical isolates of MDR *E. coli* to provide evidence of the use of chitosan-coated AgNPs as new potential agents against bacterial infections.

## Results and Discussion

### Synthesis and Characterization of SH-Cs-AgNPs

**Figure 1:**
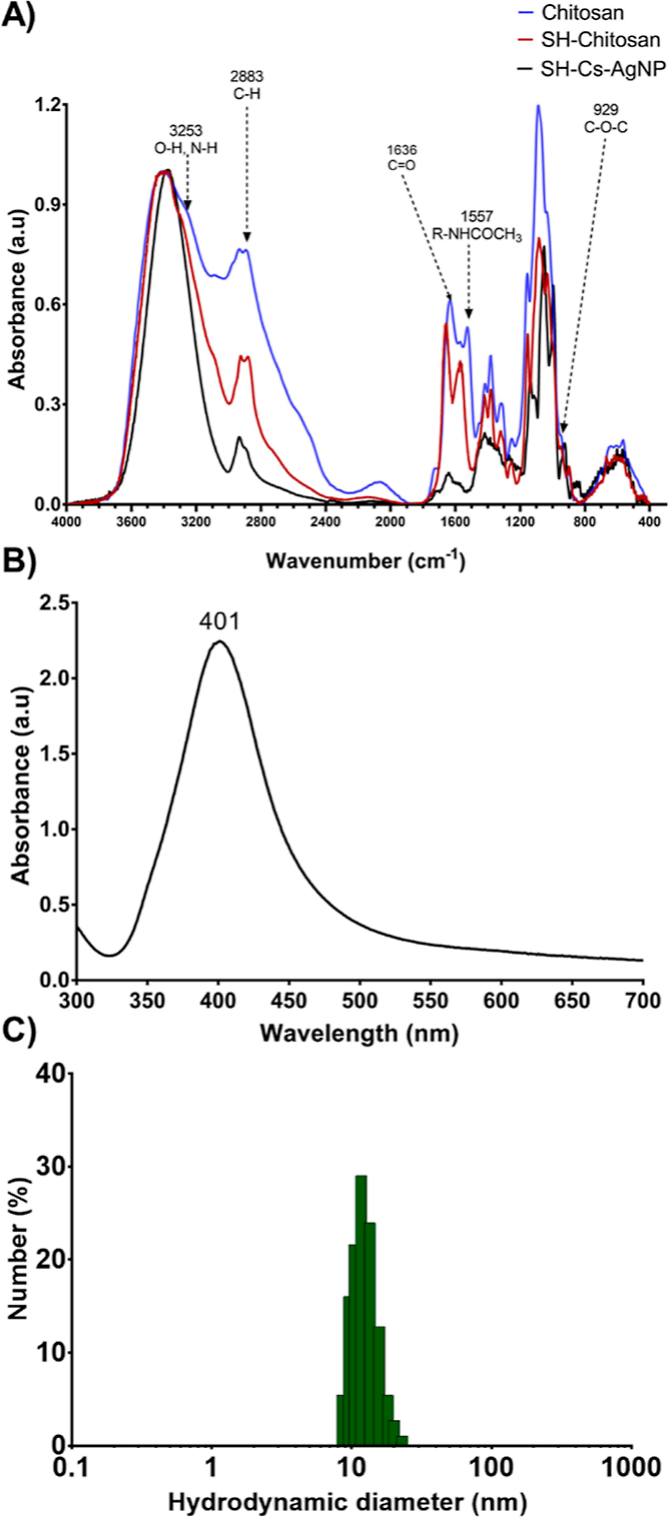
(A) FTIR spectra of chitosan, thiolated chitosan, and SH-Cs-AgNPs. (B) UV–vis spectrum of SH-Cs-AgNPs. (C) Particle size distribution of SH-Cs-AgNPs by DLS.

To facilitate the interaction between chitosan and AgNPs, chitosan was modified with thiol groups (SH-chitosan) by using mercaptopropionic acid. Modification of chitosan was demonstrated by FT-IR analysis as shown in Figure 1a. It can be observed reductions of the bandwidths at 3253 cm^–1^ and on the band intensity at 1557 cm^–1^ corresponding to the amine and amide groups due to formation of new amide bonds upon addition of the thiol groups. The degree of substitution of chitosan with mercaptopropionic acid was of 5.46% based on its acetylated proportion after the carbodiimide reaction and calculated by the absorbance area ratio (*A*_1320/1340_) of these peaks.^11^

Upon modification of native chitosan, the AgNPs were synthesized by the chemical reduction method, using SH-chitosan as a stabilizer. Upon successful formation of colloidal chitosan-coated AgNPs (SH-Cs-AgNPs), these were characterized by UV–vis spectroscopy, FT-IR, dynamic light scattering (DLS), and TEM and SEM–EDS analysis. The FT-IR of SH-Cs-AgNPs presented in Figure 1a shows the characteristic spectrum related to chitosan, demonstrating its deposition onto the surface of AgNPs.

The UV–vis spectra in Figure 1b confirmed the presence of AgNPs by denoting their characteristic localized surface plasmon resonance (LSPR) band at 401 nm. The size of the SH-Cs-AgNPs was obtained by dynamic light scattering technique (see Figure 1c) and presented as number (%) of nanoparticles, where the hydrodynamic diameter was 14.9 ± 3.8 nm (PDI: 0.090) with a zeta potential of 18.0 ± 2.0 mV, suitable to achieve a good colloidal stability by electrostatic interaction [if required, size distribution by intensity and volume (%) can be observed in Figure S1].

The presence of SH-Cs-AgNPs was studied by SEM-EDS analysis, where the presence of a peak at 3 keV is characteristic of crystalline silver, and the presence of carbon and oxygen relates to the chitosan coating of the AgNPs (Figure S2); meanwhile, in the SEM images we can observe the chitosan coating of AgNPs (white arrows). Furthermore, the nanoparticles were stable in colloidal solution for up to 288 h in different media and pH (Figure S3).

**Figure 2:**
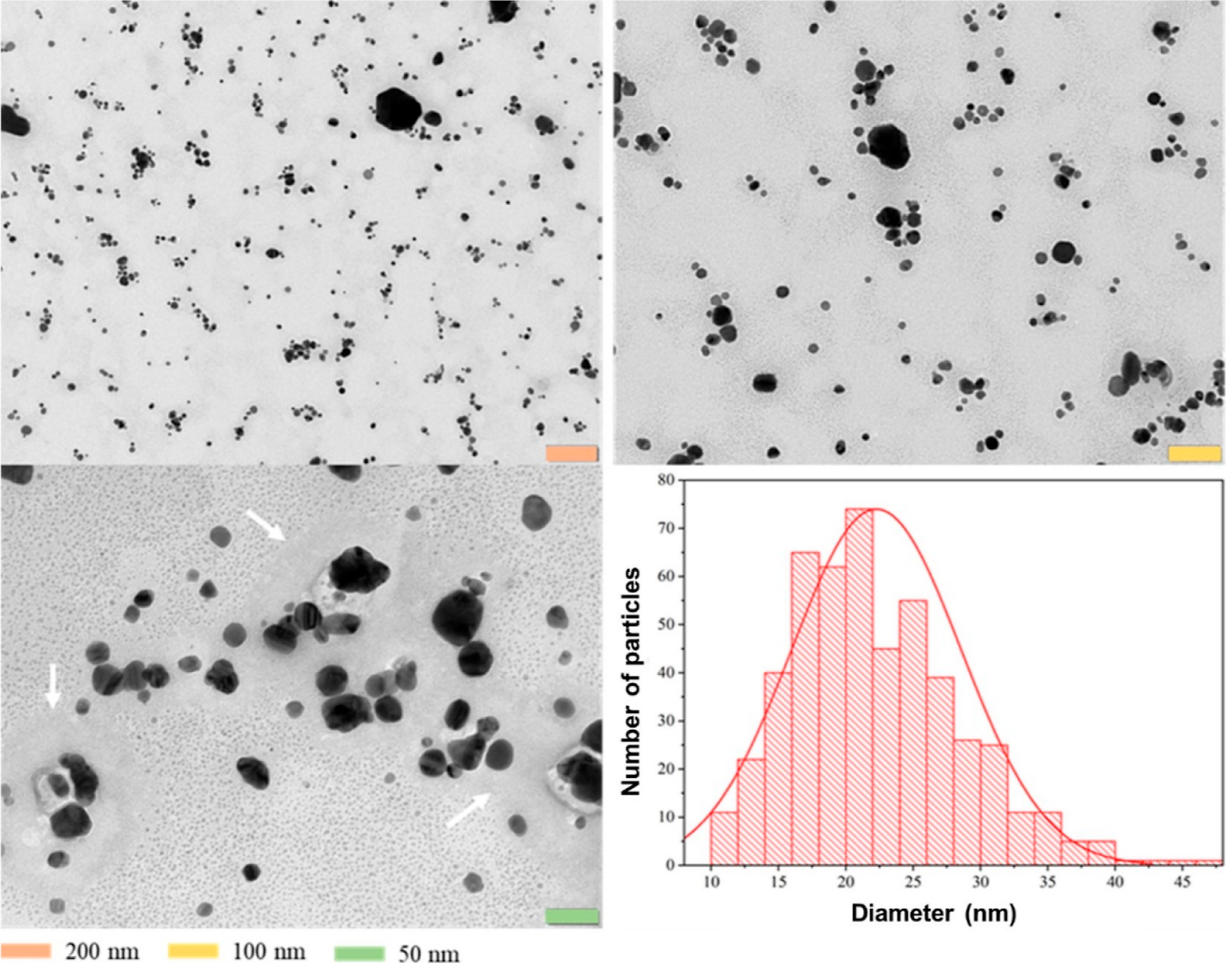
TEM images and size distribution of SH-Cs-AgNPs. White arrows represent the SH-chitosan polymeric matrix.

The morphology and size of SH-Cs-AgNPs was determined by transmission electron microscopy (TEM), since it is the gold standard for characterization of inorganic nanoparticles. Representative images and size distributions are presented in Figure 2. The SH-Cs-AgNPs had a spherical morphology; the white arrows indicate areas of lower electronic density corresponding to the chitosan surface. The size of SH-Cs-AgNPs was of 17.80 ± 2.67 nm, similar to that obtained by DLS.

### SH-Cs-AgNPs Exhibit Antibacterial Activity Against UPEC

**Figure 3:**
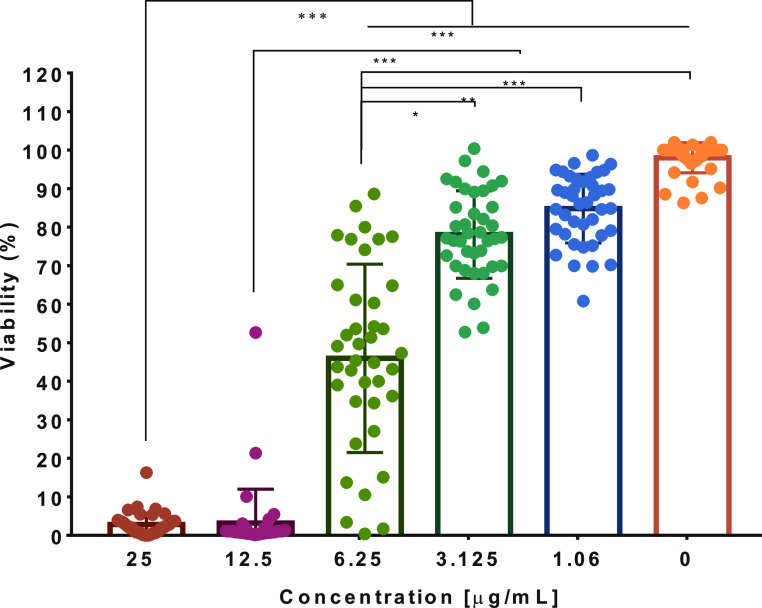
UPEC clinical isolates treated with different concentrations of SH-Cs-AgNPs (25 to 0 μg/mL). Each point represents a UPEC strain (*n* = 40). Viability of each strain reflects the average of three independent experiments ± standard deviation. ***(*p* < 0.0001); **(*p* < 0.001); *(*p* < 0.05). The graph shows the antibacterial effect of SH-Cs-AgNPs on the entire population of clinical isolates, and the standard deviation for each nanoparticle concentration is influenced by the individual viability percentages of each strain. Statistical analysis was performed by two-way ANOVA Tukey’s multiple comparison test.

To assess the antibacterial activity of SH-Cs-AgNPs against 40 clinical isolates of UPEC, the determination of the minimum inhibitory concentration (MIC) and minimum bactericidal concentration (MBC) was performed (Table S2). The results in Figure 3 show the general viability of all UPEC strains treated with SH-Cs-AgNPs, while in Figure S4 the viability of each isolate post treatments is shown; it can be observed that SH-Cs-AgNPs were able to completely inhibit the growth of almost all UPEC strains at the concentrations of 25 and 12.5 μg/mL. Nevertheless, variations in viability between strains were observed at 6.25 μg/mL, which could be associated with virulence or resistance characteristics of each strain. Of the 40 UPEC strains, 2.5% (*n* = 1) had a MIC of 25 μg/mL, 15% (*n* = 6) presented a MIC of 6.25 μg/mL, and 82.5% (*n* = 33) a MIC of 12.5 μg/mL. The MBC was coincident with the MIC values (Table S2). Our MIC values are even lower than those established by the Clinical Laboratory Standard Institute (CLSI, 2023) for some first-choice antibiotics for the treatment of UTI, such as fosfomycin (≤64 μg/mL) and nitrofurantoin (≤32 μg/mL),^12^ and are comparable with other first-choice antibiotics, considering that most of the strains evaluated are MDR. Fosfomycin and nitrofurantoin are two of the antibiotics recommended in the treatment of UTI, and so far, in Mexico, they represent two of the most efficient therapeutic options.^3^ In this sense, we determined the MIC of fosfomycin for the 40 clinical isolates (Table S2), and we observed that eight were considered as nonsusceptible (resistant or with intermediate resistance) with MIC values of 128–256 μg/mL, that represent 10 times the amount of SH-Cs-AgNPs evaluated in this work, thus reinforcing our conclusion that nanoparticles could represent an important therapeutic option in the future. In the case of nitrofurantoin, only two strains (UPEC 34 and 35) were resistant (Table S1); nevertheless, both are MDR strains.

**Figure 4:**
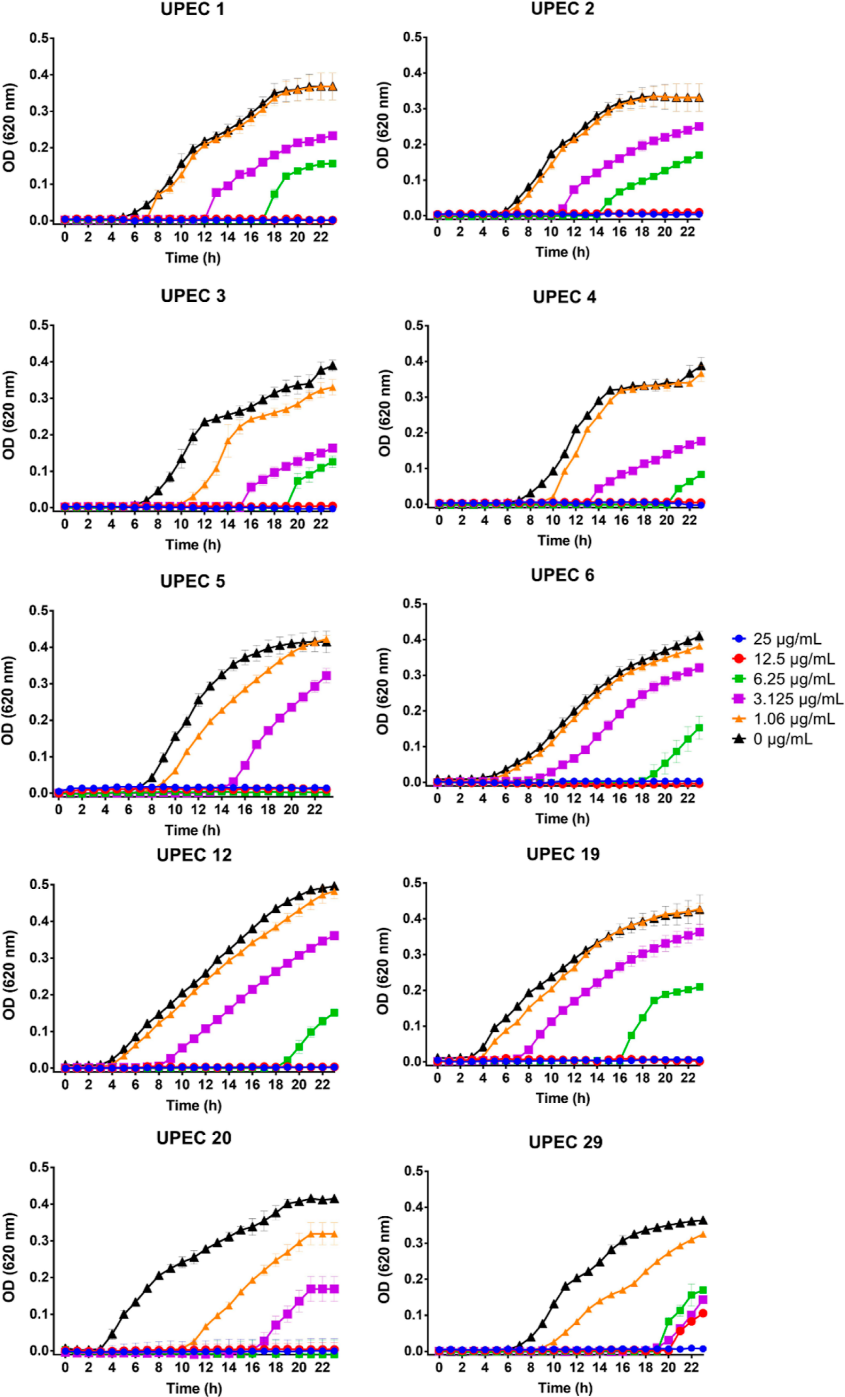
Growth curves of UPEC clinical isolates treated with different concentrations of SH-Cs-AgNPs (25 to 0 μg/mL). Data reflect the average of three independent experiments ± standard deviation.

In accordance with these results, 10 strains were selected, based on the obtained MIC values and their virulence characteristics, such as adherence, biofilm formation, and antibiotic resistance, as bacterial models to evaluate the potential of SH-Cs-AgNPs to inhibit the survival and pathogenic mechanisms of these bacteria. The MIC of SH-Cs-AgNPs against the selected isolates, except for UPEC 29, was 12.5 μg/mL. The growth curve of all isolates showed a delay, especially at concentrations of 3.12 and 6.25 μg/mL (Figure 4). These results are in accordance with previous studies demonstrating potent activity of chitosan-functionalized AgNPs on MDR clinical isolate, although the number of strains was limited, and no virulence characteristics were reported.^7^ Interestingly, UPEC 29 was found to be the most resistant to the bactericidal effect of SH-Cs-AgNPs, exhibiting a significant delay in the microbial growth curve at 12.5 μg/mL. This isolate belongs to phylogenetic group B1 and has low virulence traits (*fimH*, *fliCD*, *iha*, and *feoB*) and moderate biofilm production, but interestingly this isolate was identified as a carbapenemase and extended spectrum β-lactamase (ESBL) producer and positive for expression of *bla*_CTX-M_ and *bla*_TEM_ genes (data not shown). Interestingly, this delay on the growth curve for these strains was observed in our previous work upon treatment with propolis-AgNPs.^13^ It is not clear if antibiotic resistance mechanisms could be responsible for resistance against AgNPs; nevertheless, there is evidence of a coselection of antibiotic resistance genes (mainly *bla*_CTX-M_) and heavy metal resistance genes that could explain the variability on the susceptibility of different MDR strains against AgNPs.^14^ These findings reinforce the need to evaluate these nanosystems in a large number of bacterial strains with different virulence and resistance characteristics and, thus, design new nanosystems that are able to evade these resistance mechanisms.

### Cytotoxic Activity of SH-Cs-AgNPs

The cell viability of HeLa cells treated with SH-Cs-AgNPs was determined to standardize the concentration of SH-Cs-AgNPs to be used in the posterior adherence assays. The concentrations tested were ranged from 12.5 to 100 μg/mL. The SH-Cs-AgNPs were not toxic to HeLa cells, showing a 100% cell viability even at 100 μg/mL; a four times higher concentration than that evaluated for the antibacterial activity (Figure S5).

### Antibiofilm Activity of SH-Cs-AgNPs

**Figure 5:**
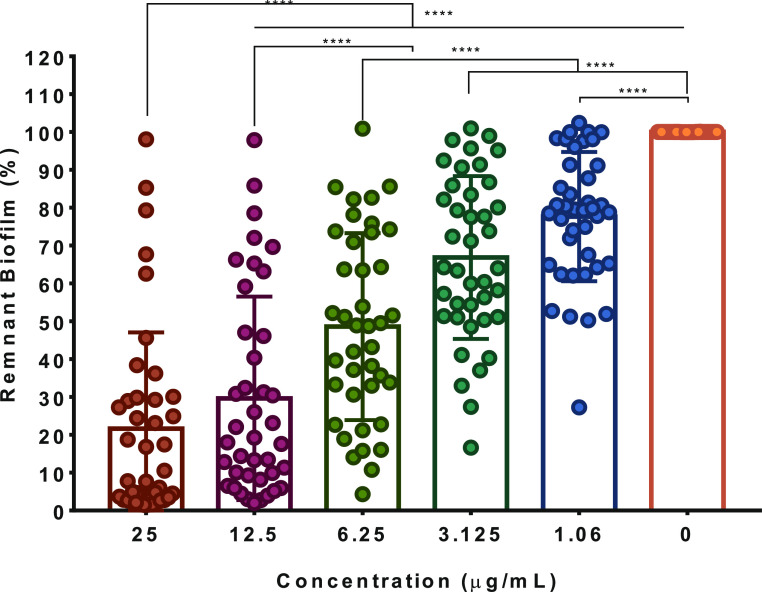
Antibiofilm activity of different concentrations of SH-Cs-AgNPs against UPEC clinical isolates. Each point represents a UPEC strain (*n* = 40). Data reflect the average of three independent experiments ± standard deviation. ****(*p* < 0.0001). The graph shows the antibiofilm effect of SH-Cs-AgNPs on the entire population of clinical isolates, and the standard deviation for each nanoparticle concentration is influenced by the individual remanent biofilm percentages post treatment of each strain. Statistical analysis was performed by two-way ANOVA Tukey’s multiple comparison test. The outliers are due to the heterogeneity of the isolates; however, to avoid overestimation, these data were omitted from the statistical analysis.

Figure 5 shows the effect of the different concentrations of SH-Cs-AgNPs on the reduction of the amount of preformed biofilm in the total population of analyzed clinical isolates, while, in Figure S6, the result for each isolate is observed. As expected, the effect of nanoparticles on the preformed biofilm presented variations in some of the strains, this could be attributed to their genetic characteristics; however, in 83% of the analyzed clinical isolates, a reduction in the amount of preformed biofilm with respect to the concentration of SH-Cs-AgNPs was observed, being more evident at concentrations of 25 and 12.5 μg/mL (*p* < 0.0001).

In addition, 36 of the 40 strains presented a reduction of the biofilm matrix upon treatment with SH-Cs-AgNPs; meanwhile, isolates UPEC 7, UPEC 12, UPEC 14, UPEC 15, UPEC 22, UPEC 29, and UPEC 31 were more resistant to the nanoparticle activity compared to the other strains, with a remanent biofilm post treatment higher than 50% even at the highest concentration implemented. Interestingly, there was no correlation between their virulence and resistances characteristics. Nevertheless, all strains (except UPEC 22) were classified as MDR and harbored genes such as *bla*_CTX-M_ or *bla*_TEM_. Also, one of them (UPEC 7) is a carbapenemase producer, while UPEC 29 is an ESBL producer. We suspect that the poor antibiofilm activity exhibited by the nanoparticles on these isolates could be due to the expression of genes involved with heavy metal-induced efflux pumps since their coexistence with genes related to ESBLs (*bla*_CTX-M_ and *bla*_TEM_, which are present in our four isolates) has been reported in the same plasmid.^15,16^ However, it is necessary to deepen in the probable mechanisms involved in resistance of bacterial biofilms to antimicrobial agents.

### Antiadherence Activity of SH-Cs-AgNPs

**Figure 6:**
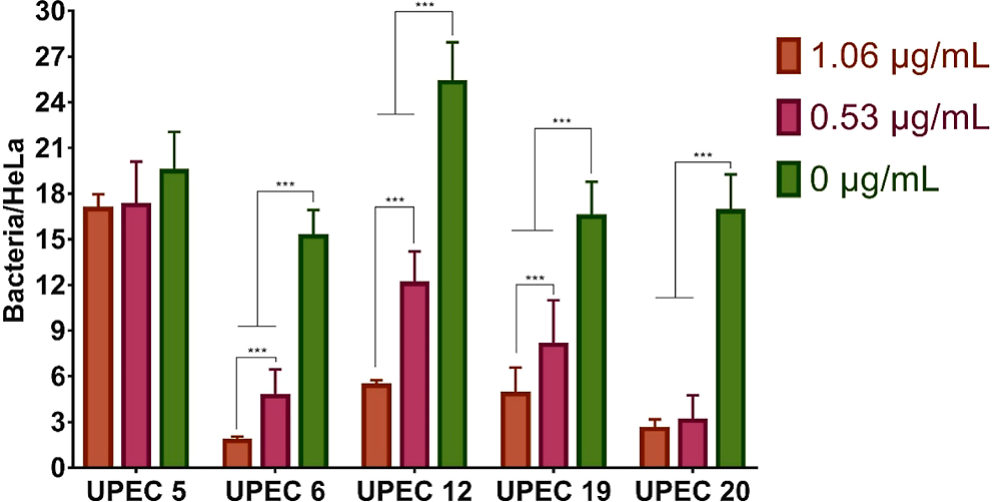
Antiadherence activity of SH-Cs-AgNPs on hyperadherent clinical isolates of UPEC. Data reflect the average of three independent experiments ± standard deviation. ***(*p* < 0.0001). Statistical analysis was performed by two-way ANOVA Tukey’s multiple comparison test. A mean of four hundred cells were counted by brightfield microscopy at 40× objective.

Bacterial adherence to cells is the first step toward the establishment of infection; hence, it is important for new antibacterial agents to be able to reduce bacterial adherence to promote their elimination, especially in UTIs. For this assay, five hyperadherent strains of UPEC were selected based on previously reported results (UPEC 5, 6, 12, 19, and 20).^17^ The concentrations of SH-Cs-AgNPs evaluated were subinhibitory based on their antibacterial activity (1.06 to 0.53 μg/mL) (Figure 4). As shown in Figure 6, all strains with the exception of UPEC 5 presented a significant reduction in the number of adhered bacteria per HeLa cell, which depended on the SH-Cs-AgNPs concentration.

**Figure 7:**
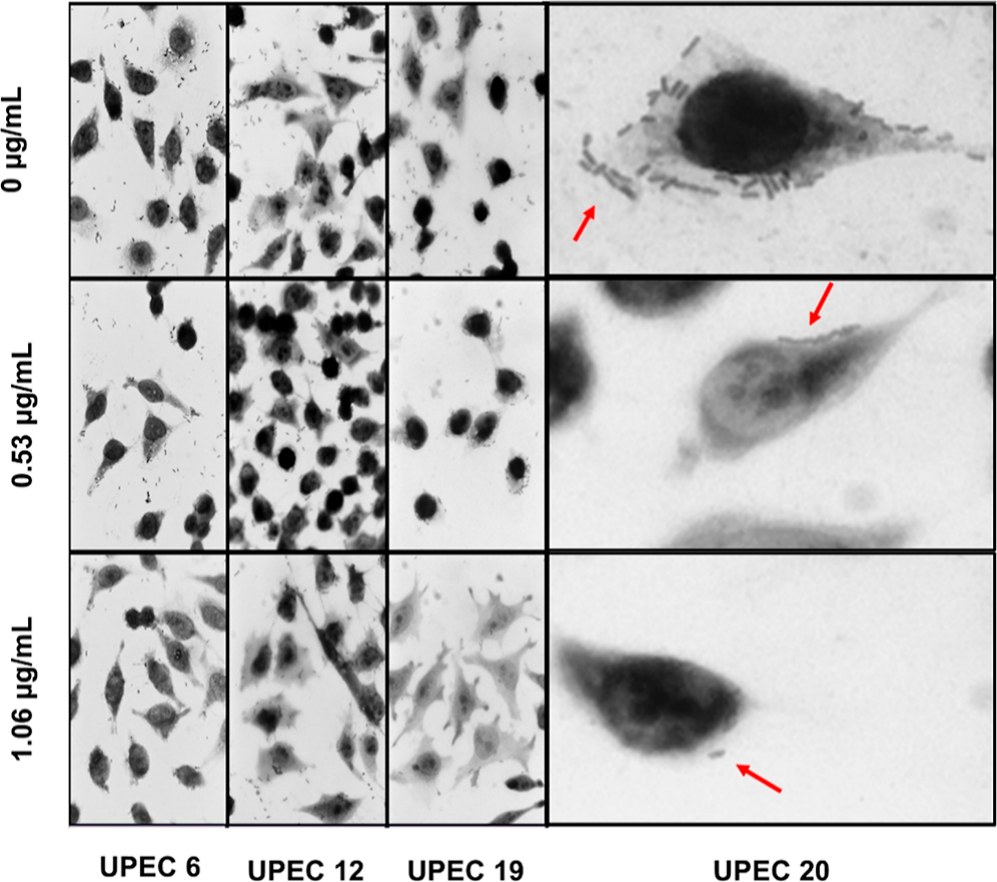
Antiadherence activity of SH-Cs-AgNPs on hyperadherent clinical isolates of UPEC and HeLa cells. Images were taken by brightfield microscopy at 40× objective. The red arrows point to the bacteria (UPEC 20) adhered to the cells.

Interestingly, the five selected strains, with exception of UPEC 5, presented more than one adherence pattern, these being localized, bricks in tandem, diffuse, and aggregative patterns (Figure S7). As shown in Figure 7, the localized, bricks in tandem, and diffuse adherence patterns were less evident upon treatment with SH-Cs-AgNPs. However, the aggregative adherence pattern remained intact at any particle concentration evaluated. UPEC 5 presented this pattern, which is associated with the expression of the adhesin AAF-1, and this adhesin is constituted by a positively charged subunit that is maintained even at pH above 9.0.^18^ Considering the net charge of our nanosystem, which is also positive, leads us to believe that the potential for our system to interact with AAF-1 is low. This could explain the null antiadhesion effect specifically in this isolation.

The ability of *E. coli* to adhere to both epithelial cells and abiotic surfaces is attributed to a variety of virulence mechanisms, including the *E. coli* common pilus (ECP), P fimbriae, Type 1 fimbriae, and FimH adhesin.^19^ These structures have been targeted in various studies aimed at reducing the infection caused by different pathotypes of *E. coli*.^20^ These findings corroborate previous reports demonstrating the ability of chito-oligosaccharides to decrease the cell adherence of *E. coli*.^21^ Additionally, AgNPs have been shown to downregulate the expression of the *fimH* gene.^22^ It is possible that the observed effect of SH-Cs-AgNPs stems from a synergistic effect between the silver itself and the oligochitosan located on the surface of the nanoparticles. However, the exact mechanism by which adherence is affected by SH-Cs-AgNPs still needs to be further assessed. FimH is a key adhesin involved in the invasion and internalization of UPEC, and it contains a carbohydrate-binding domain (CBD) capable of recognizing mannosylated uroplakins in epithelial cells.^23^ We hypothesize that the CBD may interact with oligochitosan, reducing its interaction with cells. Moreover, the negatively charged mannose-binding pocket of the CBD may interact with the positively charged SH-Cs-AgNPs.

**Figure 8:**
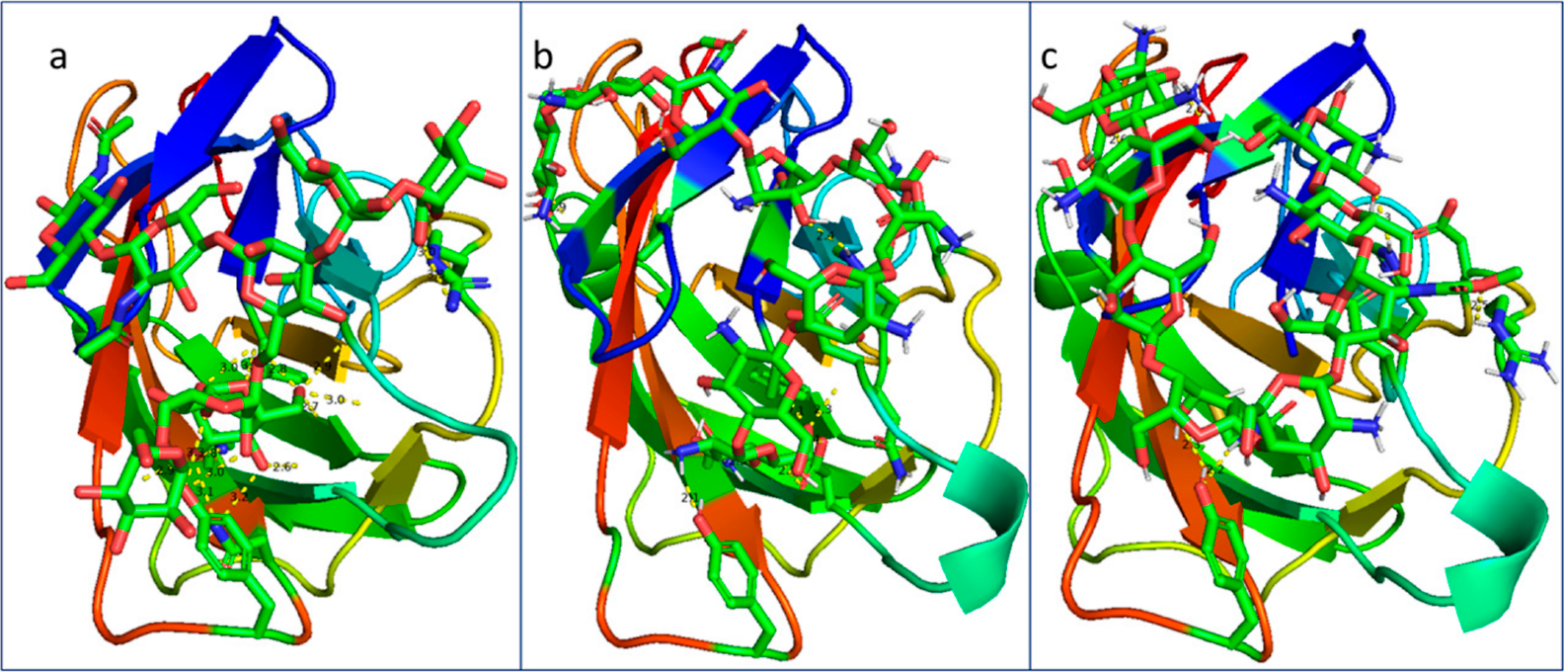
Docking interactions of FimH adhesin. (A) 3D diagram of the interactions between FimH and six-residue oligomannose. (B) 3D diagram of the interactions between FimH and oligochitosan. (C) 3D diagram of the interactions between FimH and thiolated oligochitosan.

**Figure 9:**
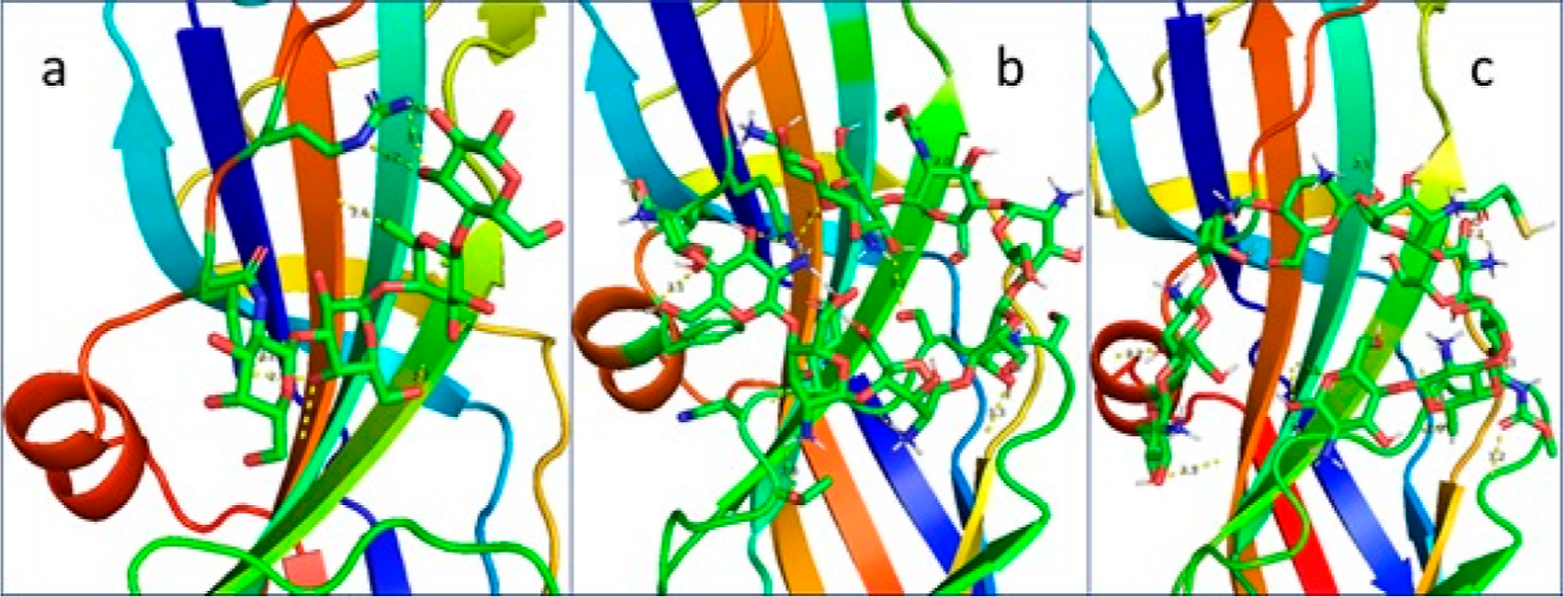
Docking interactions of PapG adhesin. (A) 3D diagram of the interactions between PapG and receptor Gb04. (B) 3D diagram of the interactions between PapG and chitosan. (C) 3D diagram of the interactions between PapG and thiolated oligochitosan.

To further clarify this statement, we carried out an in silico analysis of the interactions of FimH and PapG adhesins with chitosan and thiolated chitosan by comparing the scored binding energy with their usual ligands, a six-residue oligomannose and glycolipid Gb04, respectively. The binding energy recorded between oligochitosan and/or thiolated oligochitosan and the active site of FimH and PapG adhesins is similar to the binding energies recorded for the common ligands, oligomannose for FimH and Gb04 for PapG. The binding energy of FimH with oligomannose, oligochitosan, and thiolated oligochitosan was −5.12, −5.22, and −5.37 kcal/mol, respectively, while the association energy of the same ligands with PapG resulted in −5.19 (Gb04), −5.15, and −5.34 kcal/mol. These results suggest that chitosan can bind to the binding site of each protein preventing bacteria from anchoring to eukaryotic cell receptors, inhibiting cell invasion (Figures 8 and 9).

However, *in vivo* experiments are required to gain further insights into the molecular mechanism underlying the reduction in adherence.

## Conclusions

In summary, we synthesized chitosan-coated AgNPs using thiol-modified chitosan as the stabilizing agent for its antibacterial properties. The SH-Cs-AgNPs show a spherical shape and a size of around 17.80 ± 2.67 nm and zeta potential of 18 ± 2 mV. The SH-Cs-AgNPs inhibit growth, biofilm, and adherence to epithelial cells of clinical isolates of UPEC with a wide variety of virulence and resistant characteristics. The nanoparticles also had low toxicity against mammalian cell lines, demonstrating its possible use in *in vivo* models. The SH-Cs-AgNPs have significant potential as an antibacterial agent not only for its bactericidal activity but also for its ability to inhibit bacterial adherence to the epithelium. Further research is needed to identify the specific mechanisms by which SH-Cs-AgNPs exert these effects and their interactions with the multiple virulence factors involved in adherence and biofilm formation, as well as to observe if SH-Cs-AgNPs can reduce the adherence of UPEC in in vivo models of UTI.

## Methods

### Chemical Reagents

Chitosan oligosaccharide lactate (*M*_w_: 5000 Da), silver nitrate (>99%), sodium hydroxide (NaOH), sodium citrate (Na_3_C_6_H_5_O_7_), sodium borohydride (NaBH_4_), dimethylformamide (DMF), fetal bovine serum, bovine serum albumin (BSA), Mueller–Hinton broth, Mueller–Hinton agar, TBS broth, Dulbecco’s modified Eagle media, l-asparagine (98%), penicillin–streptomycin solution (1000 U/1 per mL), l-arginine monohydrochloride (>98%), sodium pyruvate solution (100 mM), l-glutamine solution (200 mM), *N*-hydroxysuccinimide (NHS), 1-ethyl-3-(3-(dimethylamino)propyl) carbodiimide (EDAC), and 3-mercaptopropionic acid were purchased from Sigma-Aldrich, USA. HeLa cell line (Human cervix adenocarcinoma) was purchased from the American Type Culture Collection (ATCC; Rockville, MD, USA).

### UPEC Strains

The microorganisms used for this study were clinical isolates of UPEC obtained from patients with UTI and were previously characterized by their virulence and resistance genotypes and phenotypes^17^ (Table S1). The strains were obtained with the approval of the ethical committee from Universidad de Sonora (CEI-UNISON) (registry number 07.2019, 12 March 2019).

### Synthesis of Thiolated Chitosan

Chitosan was modified with thiol groups to improve its deposition onto the surface of the AgNPs. In brief, 21 mL of chitosan (0.2 mM) were prepared in HCl (1%), and the pH was adjusted to 4.0 with NaOH (1 M) and stirred overnight. On the following day, we prepared three solutions: (1) a solution of 48 μL of 3-mercaptopropionic acid in 2 mL of dimethylformamide (DMF); (2) a solution of 107.4 mg of 1-ethyl-3-(3-(dimethylamino)propyl)-carbodiimide (EDAC) in 1 mL of DMF; and (3) a solution of 64.5 mg of NHS in 1 mL of DMF. Solutions 1 and 2 were mixed, and the resulting mixture added to solution 3, which was then mixed with 1 mL of DMF. The resulting solution was subsequently added to the chitosan one in hydrochloric acid and left to agitate overnight in the absence of light. The pH was adjusted to 9.0 by adding NaOH (1 M), which resulted in precipitation of the modified chitosan. The precipitate was washed three times with MilliQ water to remove any remaining reaction mixture, resuspended in 5 mL of water, and subjected to freezing at −70 °C for 24 h before being lyophilized and stored for further use. The modification of chitosan was confirmed by Fourier transform infrared spectroscopy (FT-IR), using a PerkinElmer Spectrophotometer (Waltham, MA, USA) at 4 cm^–1^ and a wavenumber range of 400–4000 cm^–1^.

### Synthesis of Chitosan-Coated Silver Nanoparticles

SH-Cs-AgNPs were synthesized following a chemical reduction method.^24−26^ In brief, a solution of silver nitrate (AgNO_3_) was prepared by dissolving 2 mg/mL AgNO_3_ in Milli-Q water to a final volume of 25 mL. Then, 5 mL of a 1% (w/v) solution of sodium citrate (Na_3_C_6_H_5_O_7_) was added and stirred for 1 min. Next, 312 μL of a 10 mM solution of sodium borohydride (NaBH_4_) was added, resulting in an instant deep brown colored solution. The reaction was maintained for 20 min, after which 25 mL of thiolated chitosan (0.3 mg/mL) was added, and the reaction continued for another 20 min. The resulting SH-Cs-AgNPs were stored at room temperature until further use.

### Characterization of Chitosan-Coated Silver Nanoparticles

The presence of LSPR was determined by UV–vis spectroscopy. SH-Cs-AgNPs were centrifuged at 13,000*g* for 30 min at 4 °C, the supernatant was discarded, and the pellet was redispersed in Milli-Q water. The absorption spectra ranging from 300 to 700 nm were measured by using a microplate reader (MultiskanGo, Thermo Scientific, Waltham, MA, USA). The FT-IR spectrum of SH-Cs-AgNPs was recorded by using a PerkinElmer spectrophotometer at 4 cm^–1^ and a wavenumber range of 400–4000 cm^–1^.

The hydrodynamic diameter (*D*_h_) of AgNPs was determined using a NanoZetasizer system (Malvern Instruments Ltd., Malvern, Worcestershire, UK) with a laser light (He–Ne) vertically polarized (wavelength = 633 nm, power 2 W), and a digital correlator fixed at 173°. Measurements were carried out in water (viscosity = 0.8872 mPa·s, refractive index = 1.330) at 25 °C. Then, the *D*_h_ of AgNPs was determined by dynamic light scattering measuring the fluctuation in time of the scattered light intensity coming from the interaction of the laser light with the particles suspended in a liquid medium, which are randomly moving, Brownian motion. The digital correlator records the intensity fluctuation of the light scattering and correlates with respect to the time, which is described by the normalized intensity correlation function, *g*_2_(τ)^27^
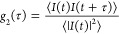


However, as experimentally it is not possible to determine the position of each particle in the scattered volume, the motion of particles relative to each other are correlated by the normalized electric field correlation function *g*_1_.^28^
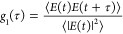
*g*_2_ and *g*_1_ are related through the Siegert equation *g*_2_ (τ) = *B* + β(*g*_1_(τ))^2^, where *B* is the baseline and β corresponds to a coherence factor.

In the case of a monodisperse sample, the exponential decay of *g*_1_(τ) is determined by a decay constant, Γ, associated with particles undergoing Brownian motion. Consequently, *g*_1_(τ) is expressed as *e*^(−Γτ)^, leading to the relation *g*_2_(τ) = 1 + β*e*^(−2Γτ)^. In contrast, within a polydisperse sample, *g*_2_ encompasses multiple exponential decay components. Consequently, there exists a summation of exponential decay rates *G*(Γ), each corresponding to individual particles within the sample.^28^
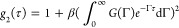
Γ is directly related to diffusion coefficient (*D*τ) as follows Γ = −*D*_τ_*q*^2^, where *q* corresponds to the magnitude of the scattering wave vector, *q* = (4π*n*_0_)/λ sin(θ/2), where λ is the wavelength, *n*_0_ is the refractive index of solvent, and θ is the angle of the detected scattered light. Then, data are analyzed by fitting the correlation function, assuming (i) a monomodal distribution approach (cumulant fitting) or (ii) a non-monomodal distribution, fitting the correlation function considering multiple decays rates. Then, particle size is determined by the Stokes–Einstein relation^29^
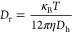
where κ_B_ is Boltzmann’s constant, *T* the absolute temperature, and η is the dynamic viscosity. *D*_h_ is the diameter of a hypothetical hard sphere that would feel the same hydrodynamic drag as the particle.

The size and shape of SH-Cs-AgNPs was determined by transmission electron microscopy by using a JEOL JEM-2010 (Jeol, Peabody, MA, USA) microscope with a voltage of 120 kV in the range of 100,000–500,000X. The samples were prepared by deposition of 50 μL of SH-Cs-AgNPs over a copper grid. The TEM images were analyzed by using the specialized software ImageJ (National Institutes of Health, Bethesda, MD, USA).^30^

### Cytotoxic (MTT) Activity

The cytotoxicity of SH-Cs-AgNPs was evaluated by the MTT assay against HeLa cells.^31^ Briefly, cells were cultivated in D5F medium until they reached 70–90% confluence. Next, 50 μL of a cell suspension (10,000 cells) were seeded into each well of a 96-well flat bottom microplate (Corning) and incubated for 24 h at 37 °C and 5% CO_2_. Then, SH-Cs-AgNPs were dispersed in D5F medium at different concentrations (100–12.5 μg/mL), and 50 μL of SH-Cs-AgNPs were added to each well; the microplate was incubated for 24 h at 37 °C and 5% CO_2_. Four hours before the 24 h incubation time, the medium was removed and then 100 μL of D5F medium was added. Next, 10 μL of an MTT solution (5 mg/mL) was added, and the microplate was incubated at 37 °C and 5% CO_2_ for 4 h. Finally, the formazan crystals were dissolved using 100 μL of DMSO, and the absorbance was measured at 570 nm using a MultiskanGo MicroPlate reader (Thermo Fischer Scientific, Waltham, USA). A wavelength of 630 nm was used as reference.

### Antibacterial Activity

The antibacterial activity of SH-Cs-AgNPs was evaluated using the broth microdilution method by the Clinical and Laboratory Standards Institute (CLSI).^12^ To prepare the SH-Cs-AgNPs, they were suspended in Mueller–Hinton media and adjusted to varying concentrations (ranging from 25 to 1.06 μg/mL). Next, 100 μL of the nanoparticles were dispensed into each well of a 96-well microplate. All strains were subcultured on Mueller–Hinton agar, and isolated colonies were adjusted to a 0.5 McFarland standard (1 × 10^8^ CFU/mL). The inoculum was then diluted 20 times, and 10 μL of the diluted inoculum was added to each well of a 96-well microplate containing the nanoparticles. For the 40 UPEC strains, the microplates were incubated for 23 h at 37 °C, and the optical density was read using a microplate reader (MultiskanGO, Thermo Fischer Scientific, Waltham, MA, USA) at 620 nm. Viability (%) was determined based on the optical density of treated bacteria vs the control, which was bacteria without nanoparticles; this analysis was performed for each strain. MIC was determined as the lowest concentration at which visible growth of bacteria was observed: meanwhile, for the MBC, after the 23 h of incubation, 10 μL of each well were plated onto Mueller–Hinton agar plates and incubated for 24 h. The results are expressed as the mean of three independent experiments in triplicate.

For the growth kinetics of the 10 UPEC strains, the same methodology was used, but the microplates were incubated for 23 h at 37 °C, and the optical density was read every hour. Selected strains and their virulence or resistance characteristics are shown in Supporting Information. The isolates were selected according to both resistance and virulence profiles, mainly the presence of genes associated with adherence at the bladder (*fimH* and *sfaD*/*focC*) and kidney (*papC*, *papG-II*, and *sfaD*/*focC*) levels as well as biofilm formation (*fimH*, *fliCD*, and *agn43*).

### Antibiofilm Assay

The assessment of the effect of SH-Cs-AgNPs against the bacterial biofilm was conducted using a previously reported method with certain modifications.^32^ On Mueller–Hinton agar, all strains were subjected to subculturing for an overnight incubation. Following this, Mueller–Hinton broth was inoculated with one colony (CFU) from each strain, and it was incubated overnight at 37 °C. The next day, a 1:14 dilution of each bacterium was prepared, and 300 μL of this dilution was introduced into individual wells of a 96-well microplate. The microplate was subsequently incubated at 37 °C for 48 h. Nonadherent bacteria were removed, and the microplate was washed three times with sterile phosphate-buffered saline (PBS). Next, each well received 150 μL of Mueller-Hinton broth containing varying concentrations of SH-Cs-AgNPs (ranging from 25 to 1.06 μg/mL), and the plate was incubated for an additional 24 h at 37 °C. Following this incubation, the supernatant was eliminated, and PBS was used to wash the microplate once more. Then, 20 μL of 0.1% (v/v) crystal violet solution was added to each well, and the plate was allowed to incubate at room temperature for 15 min. Subsequently, the microplate was washed with PBS, and 230 μL of absolute ethanol was added to dissolve the biofilm. Finally, the microplate was read at 600 nm using a MultiSkanGO MicroPlate reader (ThermoFisher Scientific, Waltham, MA, USA). The results represent the average of three independent experiments in triplicate and are expressed as the percentage of biofilm remaining post treatment compared to the untreated bacterial control for each strain.

### Antiadherence Assay

HeLa cells served as the epithelial cell model in the antiadherence assay for UPEC strains treated with SH-Cs-AgNPs. HeLa cells possess a diverse array of receptors that can interact with various UPEC-associated adhesins. Consequently, they have been extensively employed to evaluate the adherence capacity and patterns of UPEC strains.^33,34^ Five hyperadherent strains were selected based on previously reported adherence results.

To prepare HeLa cells for the experiment, we cultured them in D5F medium (comprising DMEM medium supplemented with 5% fetal bovine serum) and incubated them at 37 °C and 5% CO_2_ until they reached 70–90% confluence. Subsequently, the cells were trypsinized, counted, and adjusted to a concentration of 25,000 cells/mL. Two mL of this cell suspension were plated in each well of a 6-well microplate with coverslips and incubated for 24 h at 37 °C and 5% CO_2_.

Next, the SH-Cs-AgNPs were adjusted at different concentrations (0.53 and 1.06 μg/mL) in D5F medium and added to the respective wells. Concurrently, we adjusted a suspension of each UPEC clinical isolate to a density of 0.5 on the McFarland scale (1 × 10^8^ CFU/mL) after a 24 h preculture in Luria–Bertani broth. This adjustment was performed using a D5F medium without antibiotics.

Following these preparations, we added 15 μL of the adjusted UPEC inoculum to each well containing HeLa cells achieving a multiplicity of infection (MOI) of 30:1 (Bacteria/HeLa). After incubation, the monolayer was thoroughly washed with sterile PBS three times to remove any unattached bacteria. Subsequently, the cells were fixed with methanol for 10 min, allowed to air-dry at room temperature, and stained with Giemsa for 15 min. The coverslips were then removed, mounted on slides, and used to count the adherent bacteria per HeLa cell. To facilitate this, we employed brightfield microscopy and counted 10 fields per slide at 40× objective magnification. Results are expressed as the mean of three independent experiments performed in triplicate, with the error bar indicating the standard error of the mean. Based on the number of adherent bacteria per HeLa cell, the isolates were categorized as weak adherent (≤3 bacteria/HeLa), moderate adherent (4–7 bacteria/HeLa), or strong adherent (>8 bacteria/HeLa). Additionally, the adherence patterns of each clinical isolate were determined.

### Docking Analysis

The molecular docking of bacterial adhesins with chitosan was carried out using the AutoDock Vina software (1.1.2).^35^ The crystal structures of bacterial proteins were downloaded from the protein data bank (RCSB PDB, https://www.rcsb.org/). Bacterial FimH (8BY3) and PapG (1J8R) adhesins from *E. coli* were used as protein models of adhesion. Then, bacterial proteins were prepared by AutoDock Tools, removing water molecules and adding the polar hydrogen atoms as well as the Kollman charges. The grid parameter used for 8BY3 protein was fixed to a box size of 26 Å × 32 Å × 26 Å centered at 58.705, 18.023, and 61.038 Å, while the dimension of the grid box for 1J8R was 38 Å × 24 Å × 34 Å centered at 16.103, 13.177, and 62.133 Å. In the same way, AutoDock Tools were used to add the polar hydrogen atoms and Gasteiger charges to the ligand (oligochitosan and thiolated oligochitosan). After vina docking, the resulted complexes were processed with the PRODIGY Web server^36^ to predict the binding affinity (Δ*G*, kcal/mol)^37,38^ in the respective complexes among 8BY3 and 1J8R with native chitosan and thiolated oligochitosan, which were compared with the binding energy of the FimH-oligomanose-6 and PapG-2-acetamido-2-deoxy-β-d-galactopyranose-(1-3)-alpha-d-galactopyranose-(1-4)-β-d-galactopyranose-(1-4)-β-d-glucopyranose [Gb04] complexes.

### Statistical Analysis

Statistical analysis was performed by using GraphPad Prism 7.0. The *p* values were obtained by two-way ANOVA with the multiple comparison Tukey test.

## Supporting Information Available

Phenotypic and genotypic characteristics of analyzed clinical isolates of MDR UPEC; hydrodynamic diameter of SH-Cs-AgNPs as obtained by dynamic light scattering; SEM–EDS analysis of SH-Cs-AgNPs; colloidal stability of SH-Cs-AgNPs in different media; MIC and MBC of clinical isolates of UPEC treated with SH-Cs-AgNPs; UPEC clinical isolates treated with different concentraciones of SH-Cs-AgNPs; cytotoxic activity of SH-Cs-AgNPs on HeLa cells; antibiofilm activity of different concentrations of SH-Cs-AgNPs against UPEC clinical isolates; and adherence patterns of UPEC strains (PDF)

## Abbreviations used

AgNPs: silver nanoparticles
ATCC: American Type Culture Collection
CFU: colony forming unit
D5F: Dulbecco’s modified Eagle medium supplemented with 5% of fetal bovine serum
DLS: dynamic light scattering
DMEM: Dulbecco’s modified Eagle medium
ESBL: extended spectrum betalactamase
FT-IR: Fourier transformed infrared spectroscopy
IBCs: intracellular bacterial communities
LSPR: localized surface plasmon resonance
MBC: minimum bactericidal concentration
MIC: minimum inhibitory concentration
SH-Cs-AgNPs: thiol chitosan-coated silver nanoparticles
TEM: transmission electron microscopy
UPEC: uropathogenic *Escherichia coli*
UTI: urinary tract infection
