# Supplementary material for: Chitosan-Coated Silver Nanoparticles Inhibit Adherence and Biofilm Formation of Uropathogenic Escherichia coli
Source: ACS Infect Dis. 2024 Jan 29;10(4):1126–36. doi: 10.1021/acsinfecdis.3c00229 (PMC11019552; doi:10.1021/acsinfecdis.3c00229)
Supplement: Supplementary file 1 — id3c00229_si_001.pdf [file id3c00229_si_001.pdf]

Supplementary Information

Chitosan-Coated Silver Nanoparticles Inhibit Adherence and Biofilm Formation of Uropathogenic *Escherichia coli*

Pablo Mendez-Pfeiffer[1*], Manuel G. Ballesteros-Monrreal[1*], Josue Juarez[2**], Marisol Gastelum-Cabrera[2], Patricia Martinez-Flores[2], Pablo Taboada[3], Dora Valencia[1**]

[1] Departamento de Ciencias Químico-Biológicas y Agropecuarias. Universidad de Sonora. Campus Caborca. CP 83600, Sonora, Mexico.

[2] Departamento de Física. Universidad de Sonora. Campus Hermosillo. CP 83000, Sonora, Mexico.

[3] Departamento de Física de la Materia Condensada. Facultad de Física. Universidad de Santiago de Compostela. CP 15782, España.

* Both authors contributed equally to this manuscript

** Corresponding author: Dora Valencia. dora.valencia@unison.mx; Josue Juarez. josue.juarez@unison.mx

Table of Content

Table S1: phenotypic and genotypic characteristics of analyzed clinical isolates of multidrug resistant uropathogenic E. coli ……………………………S2

Figure S1: Hydrodynamic diameter of SH-Cs-AgNPs as obtained by Dynamic Light Scattering (DLS) represented as intensity (%) and volume (%)…………S4

Figure S2: SEM-EDS analysis of SH-Cs-AgNPs. White arrows represent the SH-chitosan polymeric matrix……………………………………………………………S5

Figure S3: Colloidal stability assay of SH-Cs-AgNPs in different media……..S6

Table S2: Minimum inhibitory concentration (MIC) and minimum bactericidal concentration (MBC) of clinical isolates of uropathogenic E. coli treated with SH-Cs-AgNPs…………………………………………………………………...S7

Figure S4: UPEC clinical isolates treated with different concentrations of SH-Cs-AgNPs…………………………………………………………………………S8

Figure S5: Cytotoxic activity of SH-Cs-AgNPs on HeLa cells..………………...S9

Figure S6: Antibiofilm activity of different concentrations of SH-Cs-AgNPs against UPEC clinical isolates………………………………………………………S10

Figure S7: Adherence patterns of UPEC strains…………………………………S11

Table S1. phenotypic and genotypic characteristics of analyzed clinical isolates of multidrug resistant uropathogenic *E. coli* (Ballesteros-Monrreal et al., 2021). Selected strains for kinetic growth assays are marked in red.

UPEC	PG	Virulence Profile	Resistance Profile	ESBL gene	Classification
1	B2	*fimH,papG-II,sat,hlyA,traT,iroN,fyuA,iha,feoB*	AMK,GM,CIP,NOR,LVX,AMP,CFX, CTX,AMC,AMS		MDR
2	B1	*fimH,fliCD,hlyA,iucD,iutA,feoB*	GM,CIP,NOR,LVX,TSX,AMP,CFX,CFZ, CTX,CRO,FEP,ATM,AMC,AMS,TE		XDR
3	C	*fimH,papG-II,fliCD,sat,vat,traT,agn43,iroN,iucD,fyuA, iha,feoB*	AMK, GM, TSX, AMP, CFX, CTX, CRO, AMC, AMS, TE		MDR
4	C	*fimH,papG-II,fliCD,sat,vat,traT,agn43, iroN,fyuA,iha,feoB*	AMK,GM,CFX		NMDR
5	C	*fimH,fliCD,sat,kpsM,traT,iucD,feoB*	TSX,AMP,CX,CFX,CFZ,CTX,CRO, FEP,ATM,AMC,AMS		MDR
6	B2	*fimH,fliCD,sat,vat,traT,agn43,iucD,fyuA, iha,feoB*	GM, CIP, NOR, LVX, TSX, AMP, CFX, AMC, TE		MDR
7	B1	*fimH,agn43,iutA*	AMK, GM, AMP, CFX, AMC, TE, MEM	Positive	MDR
8	NT	*fimH,fliCD,hlyA,kpsM,traT,feoB*	CFX, TE		NMDR
9	B2	*papC,fimH,sfaD/focC,papG-II,fliCD, kpsM,vat,cnf-1,iroN,iucD,fyuA,feoB*	-		NMDR
10	NT	*fimH,papG-II,fliCD,vat,traT,iroN,fyuAfeoB*	GM, AMP, CFX, CFZ, CTX, TE		MDR
11	NT	*fimH,sat,hlyA,kpsM,cnf-1,agn43,iucD,feoB*	GM, CIP, NOR, LVX, AMP, CFX, CFZ, CTX, AMC		MDR
12	E	*papC,fimH,papG-II,sat,cnf-1,traT,agn43,iroN,iucD,fyuA,iha,feoB*	AMK, GM, AMP, CFX, CTX, AMC, AMS, TE	Positive	MDR
13	NT	*fimH,papG-II,fliCD,traT,feoB*	CFX, CTX, ATM		NMDR
14	E	*fimH,kpsM,iucD,feoB*	AMK, CIP, CFX, CTX, CRO, TE		MDR
15	E	*fimH,fliCD,cnf-1,traT,agn43,iucD,iha,feoB*	GM, TSX, AMP, CFX, CTX, AMC, TE		MDR
16	B1	*fimH,fliCD,hlyA,iucD,iha,iutA,feoB*	GM, CFX, CTX, AMC		NMDR
17	NT	*fimH,papG-II,fliCD,hlyA,iucD,fyuA,iha,feoB*	AMK, GM, CFX, CTX, TE		MDR
18	B2	*fimH,papG-II,fliCD,iroN,fyuA,iutA,feoB*	AMP, CFX, CTX, AMC, AMS		MDR
19	NT	*fimH,fliCD,sat,iroN,iucD,fyuA,feoB*	AMK, CFX, CTX, AMC		NMDR
20	NT	*fimH,papG-II,fliCD,hlyA,traT,feoB*	AMK, GM, AMP, CFX, CTX,		MDR

			CRO, ATM, AMC		
21	B1	*fimH,fliCD,sat,kpsM,traT,iucD,fyuA,feoB*	AMK, TSX, AMP, CFX, CFZ, CTX, CRO, AMC, AMS		MDR
22	NT	*fimH,sat,iroN,fyuA,feoB*	AMK, GM, AMP, CFX, CFZ, CTX		NMDR
23	NT	*fimH,papG-II,fliCD,traT,iucD,feoB*	AMK, GM, AMP, CFX, CTX, CRO, AMC		MDR
24	NT	*fimH,fliCD,feoB*	AMK, GM, AMP, CX, CFX, CFZ, CTX, CRO, AMC		MDR
25	E	*fimH,fliCD,iroN,iucD,fyuA,feoB*	AMK, GM, TSX, AMP, CFX, CTX, CRO, AMC, TE		MDR
26	B2	*fimH,papG-II,fliCD,iroN,iucD,fyuA,feoB*	AMK, GM, AMP, CFX, CTX, AMC, AMS		MDR
27	E	*fimH,fliCD,sat,iroN,iucD,fyuA,feoB*	AMK, GM, CIP, NOR, LVX, TSX, AMP, CX, CFX, CFZ, CTX, CRO, ATM, AMC, MEM		MDR
28	E	*fimH,sat,hlyA,kpsM,iroN,iucD,fyuA,iha,feoB*	AMK, GM, TSX, AMP, CFX, CFZ, CTX, CRO, AMC, AMS, TE		MDR
29	B1	*fimH,fliCD,iha,feoB*	AMK, GM, CIP, NOR, LVX, TSX, AMP, CFX, CFZ, CTX, CRO, ATM, AMC, TE	Positive	MDR
30	B2	*fimH,sfaD/focC,sat,hlyA,kpsM,iroN,iucD,fyuA,feoB*	TSX, AMP, CFX, CFZ, CTX, AMC, AMS, TE		MDR
31	B1	*fimH,fliCD,iha,feoB*	GM, CIP, NOR, LVX, TSX, AMP, CX, CFX, CFZ, CTX, CRO, AMC	Positive	MDR
32	B1	*fimH,fliCD,iroN,iucD,feoB*	AMK, GM, CIP, NOR, LVX, TSX, AMP, CFX, CFZ, CTX, CRO, AMC, AMS		MDR
33	B1	*fimH,fliCD,iroN,iucD,fyuA,feoB*	AMK, GM, TSX, AMP, CFX, CTX, CRO, ATM, AMC, AMS		MDR
34	B2	*fimH,papG-II,fliCD,sat,hlyA,kpsM,vat,cnf-1,traT,iroN,iucD,fyuA,feoB*	AMK, GM, MAC, CIP, NOR, LVX, TSX, MAC, AMP, CFX, CFZ, CTX, CRO, AMC, AMS, TE		MDR
35	B1	*fimH,fliCD,hlyA,kpsM,iha,feoB*	AMK, GM, MAC, CIP, NOR, LVX, TSX, MAC, AMP, CX, CFX, CFZ, CTX, CRO, AMC, IMP		MDR
36	B1	*fimH,fliCD,hlyA,kpsM,feoB*	AMK, GM, CIP, NOR, LVX, TSX, AMP, CX, CFX, CFZ, CTX, CRO, ATM, AMC		MDR
37	B2	*fimH,fliCD,sat,hlyA,kpsM,vat,iroN,iucD,fyuA,iha,feoB*	TSX, AMP, CFX, AMC, AMS		MDR

38	B2	*fimH,fliCD,sat,hlyA,kpsM,cnf-1,iucD,fyuA,iha,feoB*	GM, LVX, TSX, AMP, CFX, FEP, ATM, AMC, AMS	MDR
39	B2	*fimH,fliCD,sat,hlyA,kpsM,vat,iucD,fyuA,iha,feoB*	AMK, GM, CIP, NOR, AMP, CFX, CFZ, CTX, CRO, AMC, AMS, TE, IMP	MDR
40	B2	*fimH,fliCD,cnf-1,iucD,fyuA,iha,feoB*	CIP, NOR, LVX, AMP, CFX, CFZ, CTX, CRO, FEP, ATM, AMC, AMS TE, IMP	MDR

fimH: Type 1 pilus adhesin; ***sfaD/focC:*** F1C pilus; ***papG-II:*** Type P pilus Adhesin allele 2; ***fliCD:*** Flagellin subunit/flagellar cap; ***hlyA:*** α-hemolysin;; ***kpsM:*** Capsular variant; ***vat:*** Autotransporter secreted toxin; ***cnf-1:*** Necrotizing cytotoxic factor; ***traT:*** Complement resistance associated protein; ***iucD:*** Aerobactin; ***iutA:*** Aerobactin receptor; ***feoB:*** Ferrous iron transporter B; ***fyuA:*** Ferric yersiniabactin uptake receptor; ***sat:*** Secreted autotransporter toxin; ***iha:*** Enterobactin***;*** ***iroN:*** Salmochelin; ***papA:*** Type P pilus; **ESBL+:** Extended spectrum betalactamase phenotype; **CAR+:** Positive carbapenemases phenotype; **CTX-M; TEM;SHV:** β-lactamases associated genes; ***qnrB* and *aac(6')-lb*:** Quinolones resistance associated genes (plasmids). **MAC:** Nitrofurantoin; **GM:** Gentamicin; **CIP:** Ciprofloxacin; **NOR:** Norfloxacin; **LVX:** Levofloxacin**; TSX:** Cotrimoxazole; **AMP:** Ampicillin; **CFX:** Cefuroxime**; CTX:** Cefotaxime; **CRO:** Ceftriaxone; **FEP:** Cefepime; **ATM:** Aztreonam; **AMC:** Amoxicillin - Clavulanic acid; **AMS:** Ampicillin-Sulbactam; **TE:** Tetracycline; **AMK:** amikacin; **CF:** Cephalothin; **CL:** Colistin; **ETP:** Ertapenem; **MEM:** Meropenem; **MDR:** Multi-drug resistant; **XDR:** Extensively drug-resistant; **NMDR:** Non multi-drug resistant. Isolates selected for microbial growth curves are indicated in red. And in red with bold the isolate UPEC 29, which was also a carbapenemase and extended spectrum beta-lactamase (ESBL) producer. **PG:** Phylogenetic Group. **NT:** Unknow phylogenetic group. Clinical isolates that presented the bla_{TEM} or $bla_{CTX\text{-}M}$ genes are indicated as positive in the ESBL genes column.

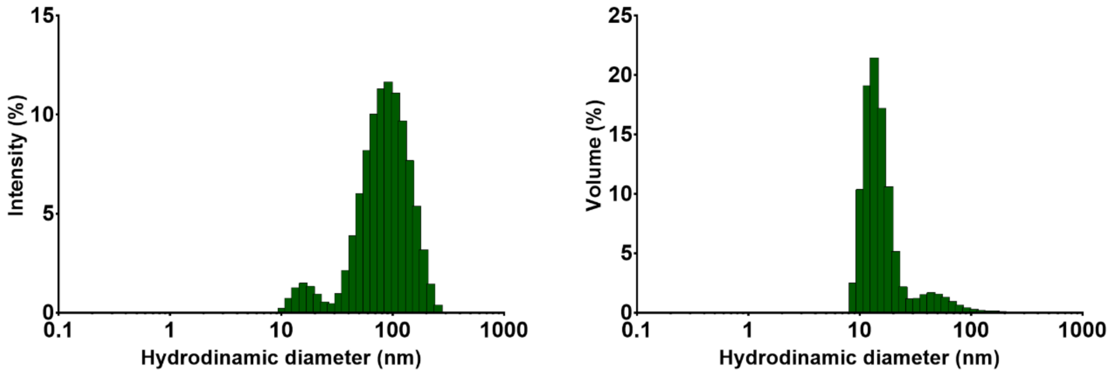

Figure S1. Hydrodynamic diameter of SH-Cs-AgNPs as obtained by Dynamic Light Scattering (DLS) represented as intensity (%) and volume (%).

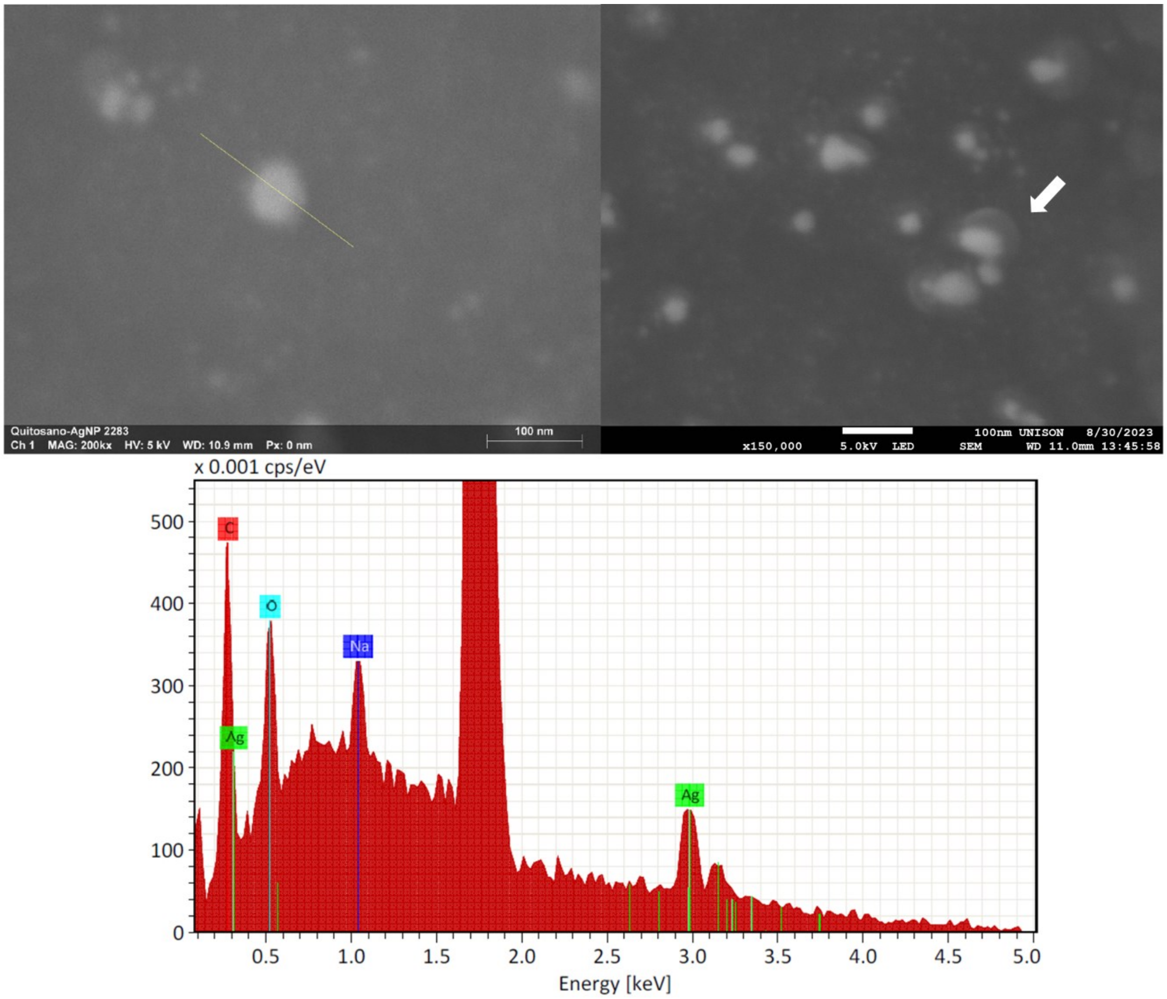

Figure S2. SEM-EDS analysis of SH-Cs-AgNPs. White arrows represent the SH-chitosan polymeric matrix.

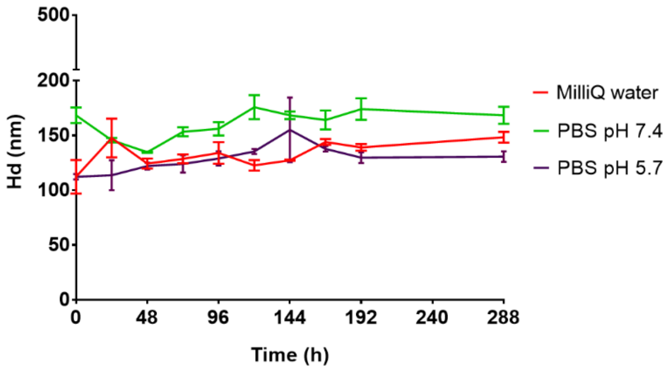

Figure S3. Colloidal stability assay of SH-Cs-AgNPs in different media. Stability was determined based on the hydrodynamic diameter measured by dynamic light scattering. Data is representative of at least three independent experiments.

Table S2. Minimum inhibitory concentration (MIC) and minimum bactericidal concentration (MBC) of clinical isolates of uropathogenic *E. coli* treated with SH-Cs-AgNPs. FOS: Fosfomycin; S: Strain sensible to Fosfomycin; R/I: Fosfomycin Non-Susceptible strain.

SH-Cs-AgNPs				FOSFOMYCIN	
**Strain**	**MIC (µg/mL)**	**MBC (µg/mL)**	**FOS Phenotype**	**MIC (µg/mL)**	**MBC (µg/mL)**
1	12.5	12.5	S	64	>256
2	12.5	12.5	R	256	256
3	12.5	12.5	R	256	256
4	12.5	12.5	S	64	128
5	12.5	12.5	S	64	256
6	12.5	12.5	S	64	64
7	6.25	6.25	S	64	64
8	6.25	6.25	S	64	64
9	6.25	6.25	S	64	256
10	12.5	6.25	S	64	128
11	12.5	12.5	R	256	256
12	12.5	12.5	S	64	64
13	12.5	12.5	S	64	64
14	12.5	12.5	S	64	128
15	12.5	12.5	S	64	64
16	12.5	12.5	S	64	128
17	12.5	12.5	R	256	256
18	12.5	12.5	R	256	128
19	12.5	12.5	S	64	64
20	12.5	12.5	S	64	128
21	12.5	12.5	S	64	64
22	12.5	12.5	I	128	256
23	12.5	12.5	S	64	128
24	12.5	12.5	S	64	128
25	12.5	12.5	S	64	64
26	12.5	12.5	S	64	64
27	12.5	12.5	S	64	256
28	12.5	12.5	S	64	64
29	25	25	S	64	64
30	6.25	6.25	S	64	64
31	12.5	12.5	S	64	128
32	12.5	12.5	R	256	64
33	12.5	12.5	S	64	256
34	12.5	12.5	S	64	128
35	12.5	12.5	S	64	64
36	12.5	12.5	S	64	64
37	6.25	6.25	S	64	64
38	12.5	12.5	S	64	128
39	6.25	6.25	S	64	64
40	12.5	12.5	I	128	128

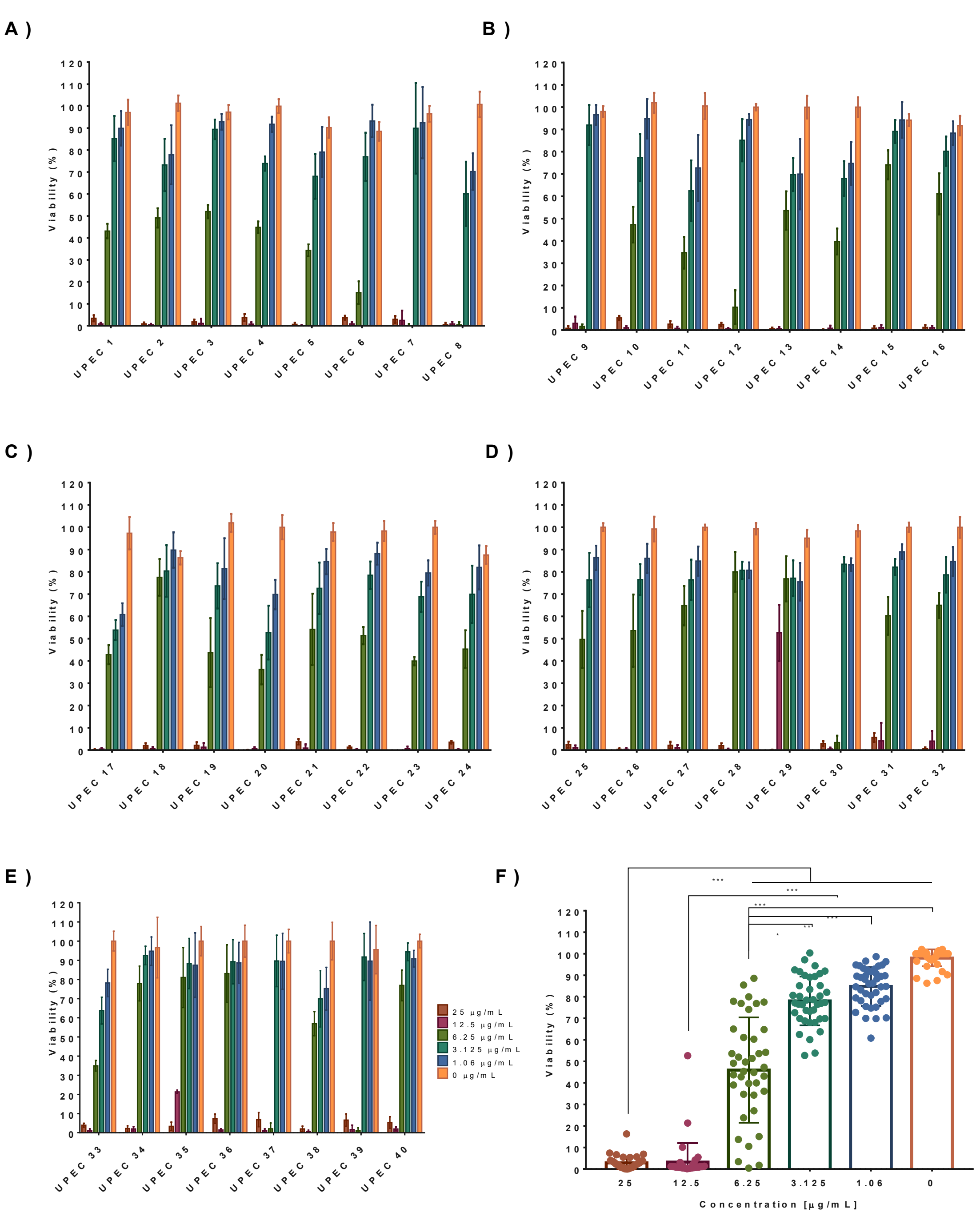

Figure S4. UPEC clinical isolates treated with different concentrations of SH-Cs-AgNPs.

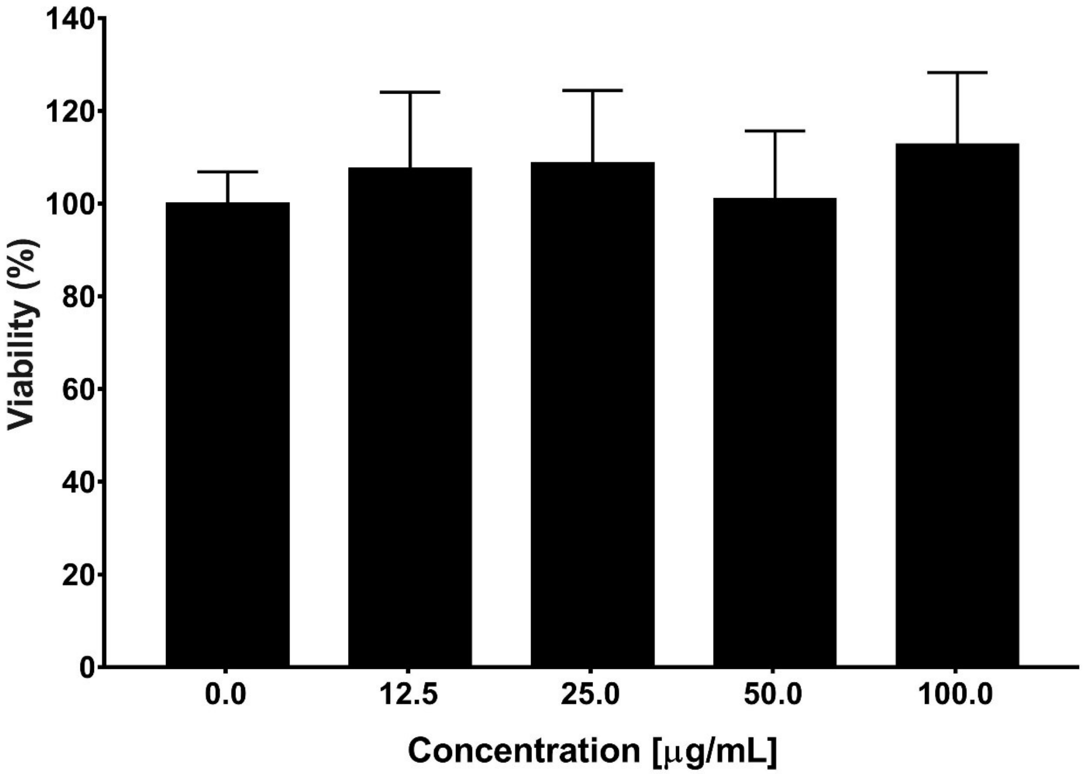

Figure S5. Cytotoxic activity of SH-Cs-AgNPs on HeLa cells. Data represents the mean of three independent experiments ± standard deviation.

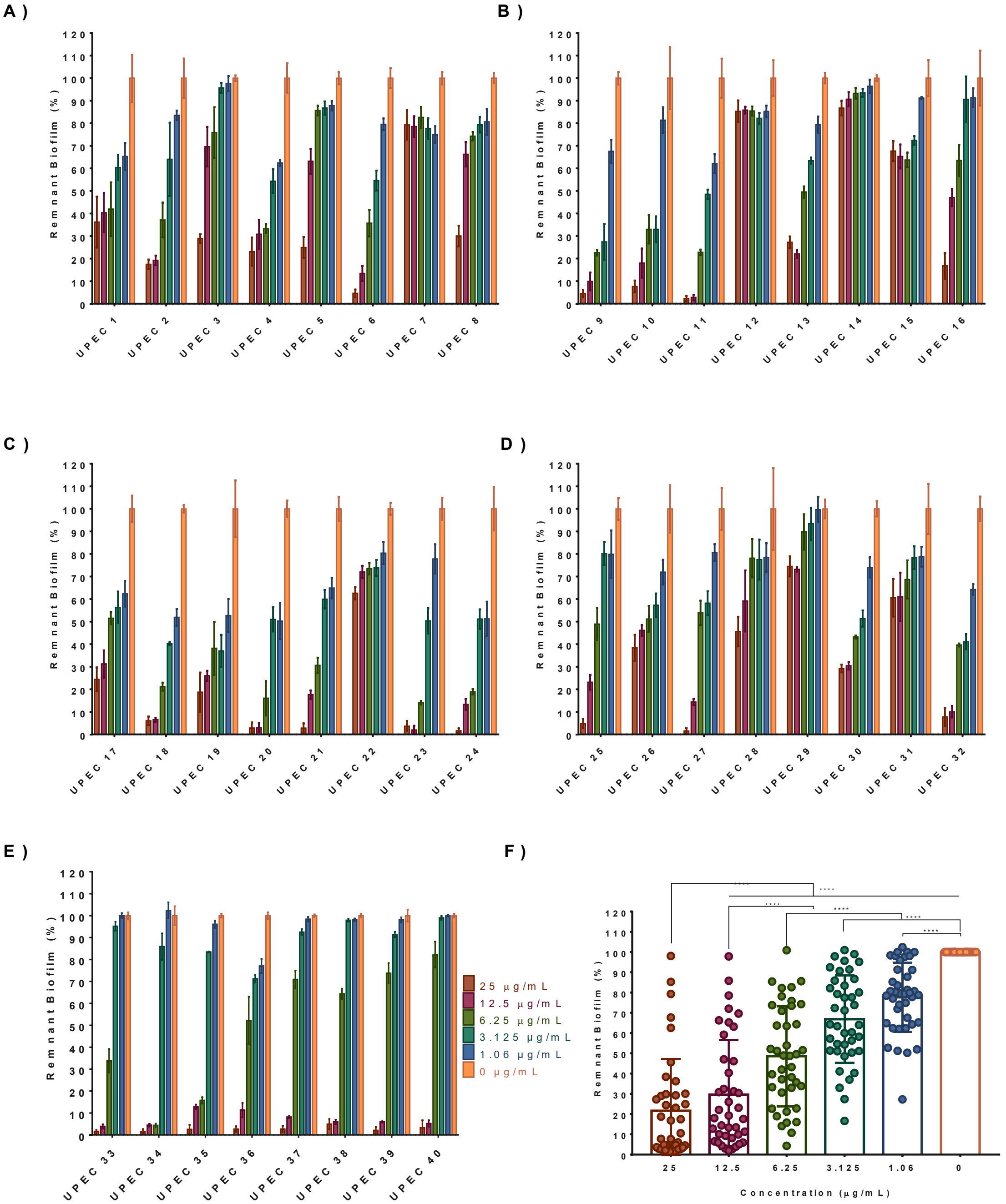

Figure S6. Antibiofilm activity of different concentrations of SH-Cs-AgNPs against UPEC clinical isolates.

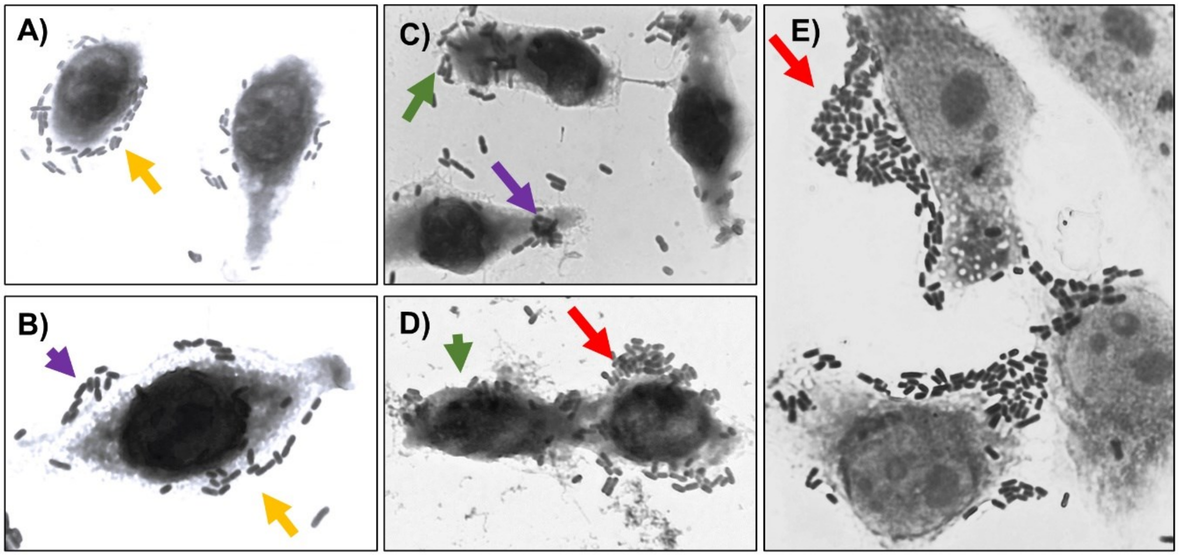

Figure S7. Adherence patterns of UPEC strains. A) UPEC 6; B) UPEC 12; C) UPEC 19; D) UPEC 20; E) UPEC 5. Bricks in tandem (yellow arrow); Localized pattern (purple arrow); Diffuse pattern (green arrow); aggregative pattern (red arrow).
